# Using Social Network as a Recruiting Tool for Research on Substance Use in the Taipei Metropolitan Area: Study Design, Implementation, and Epidemiological Estimates

**DOI:** 10.2188/jea.JE20140229

**Published:** 2015-11-05

**Authors:** Te-Tien Ting, Chuan-Yu Chen, Yu-Shu Tsai, Yen-Tyng Chen, Lien-Wen Su, Wei J. Chen

**Affiliations:** 1 Institute of Epidemiology and Preventive Medicine, College of Public Health, National Taiwan University, Taipei, Taiwan; 2 Center of Neuropsychiatric Research, National Health Research Institutes, Zhunan, Miaoli County, Taiwan; 3 Department of Public Health, College of Public Health, National Taiwan University, Taipei, Taiwan; 4 Institute of Public Health, National Yang-Ming University, Taipei, Taiwan; 5 Department of Behavioral Sciences and Health Education, Rollins School of Public Health, Emory University, Atlanta, USA; 6 Department of Addiction Science, Taipei City Hospital, Songde Branch, Taipei, Taiwan; 7 Department of Psychiatry, College of Medicine and National Taiwan University Hospital, National Taiwan University, Taipei, Taiwan

**Keywords:** respondent-driven sampling (RDS), illegal drug use, computer-assisted self-interview

## Abstract

**Background:**

This study aimed to evaluate the practical utility of respondent-driven sampling (RDS) among regular tobacco and alcohol users in Taipei, Taiwan.

**Methods:**

RDS was implemented from 2007 to 2010 to recruit seed individuals who were 18 to 50 years old, regular tobacco and alcohol users, and currently residing in Taipei. Each respondent was asked to refer up to five friends known to be regular tobacco smokers and alcohol drinkers to participate in the present study. Information pertaining to drug use was collected using an audio computer-assisted self-interview instrument. RDSAT software was used for data analyses.

**Results:**

The prevalence estimates of illegal-drug-using behaviors attained equilibrium after three to five recruitment waves. Nearly one-fifth of the participants had ever used illegal drugs, of whom over 60% were polydrug users. The RDS-adjusted prevalences of illegal-drug-using behaviors among early-onset smokers were all two or three times higher than those among late-onset smokers.

**Conclusions:**

Our results provided an empirical basis for the practicality and feasibility of using RDS to estimate illegal drug use prevalence among regular tobacco and alcohol users.

## INTRODUCTION

Illegal drug users are often clustered in social networks. Taking advantage of the underlying mechanisms (eg, the small-world theory),^1^ researchers have developed methods, such as respondent-driven sampling (RDS), to efficiently discover these people hidden in different social milieus. RDS utilizes probability-theoretical methods to compensate for nonrandom sampling and produces proper estimates to monitor risk-taking behaviors.^2^^–^^5^ However, participants’ preference and contextual constraints related to differential recruitment in the RDS recruiting process can introduce bias in chain-referral sampling. Therefore, it is important to adopt practical methods to reduce differential recruitment in implementing RDS.

In the RDS recruitment process, double incentives were used to motivate participation and harness peer approval, thereby enhancing the compliance and response rate.^6^ This practice can help decrease the occurrence of the exhaustion of the chain-referral process and increase the possibility of randomly selecting peers with similar risk-taking behaviors. In addition, a long chain of referral, in which equilibrium status can be attained, is another critical practice to ensure convergence for producing unbiased RDS estimates^7^^,^^8^; however, whether or not the minimum number of recruitment waves to assure the convergence of RDS estimates varies across populations and behaviors, particularly for extremely sensitive behaviors in some conservative societies, remains unknown. Several studies have been carried out to compare the field effectiveness of the RDS with that of other methods,^7^^–^^11^ with findings showing that certain theory-based assumptions of RDS were violated when applied in the field.^11^^–^^13^

In addition to the network clustering phenomenon, history and pattern of substance use are also important in designing research to approach illegal and polydrug users. Evidence obtained from epidemiological and clinical research has indicated that the sequences of drug experience appear nonrandom,^14^ with a majority of illegal drug users having used cigarettes^15^ or alcohol prior to the using any illegal drugs.^16^ In Taiwan, the lifetime prevalence of illegal drug use is relatively low. For example, less than 3% of a national representative sample of school-attending adolescents reported having used illegal drugs from 2004 to 2006.^17^ However, the corresponding figure among the alcohol- and tobacco-using students was much higher (approximately 24%). Given these findings, when attempting to identify illegal drug users dwelling in the community, an RDS study that targets tobacco and alcohol users may help improve efficiency. However, whether or not an RDS can be practically and feasibly used to estimate the prevalence of illegal drug use on the basis of legal substance users remains unclear.

In Taiwan, given the cultural acceptance of alcohol-containing food or beverages in most social occasions, more than 40% of high school students have ever consumed alcohol^17^; conversely, the initiation of tobacco smoking in the young population is more indicative of a deviant behavior profile. Tobacco onset age has been recognized as a strong factor in explaining the heterogeneity in subsequent risk of advanced stages of problem use (eg, nicotine dependence)^18^^–^^20^ or usage of other illegal drugs.^21^^,^^22^ Recent research has further indicated that there may be genetic predisposition to early tobacco smoking.^23^^–^^25^ It is of interest to investigate whether early onset of tobacco use may serve as a factor in explaining differential profiles of illegal drug use among RDS-recruited participants.

In this study, a series of RDS implementations was undertaken in Taiwan, where the stigma attached to illegal drug use runs high and illegal drug users are considered criminals by law.^26^^,^^27^ In the metropolitan area of Taipei, where the RDS was carried out, the percentage of illegal drug users in the adult population has been reported to be low.^28^^,^^29^ The primary aim of this study was to evaluate the practical utility of RDS among the non-institutional population and to investigate the prevalence of illegal drug use among regular tobacco and alcohol users in the Taipei metropolitan area.

## METHODS

### Design of RDS and participants

RDS uses the recruitment pattern of each participant to adjust for potential confounding in estimating the transition probability. Hence, in the design of an RDS study, it is important to have a well-specified protocol for the source of seeds and the rules of respondent recruitment and information collection on respondents’ social network. This RDS study was implemented from 2007 to 2010, and initial recruitment of seeds was conducted in two types of settings (both community-based and hospital-based seeds) in the greater Taipei area, with the assumption that regular tobacco and alcohol users may share or belong to the same “complete social network”. Through repeated samplings in each study year, prevalence estimates of illegal drug use could be nondifferentially produced. The study was approved by the institutional review board of the College of Public Health, National Taiwan University.

#### Source of seeds

Community-based seeds were diversified and recruited from several sources, such as young smokers who drank beer in nightclubs, frequent KTV party (ie, individual-room karaoke bar) attendees who used alcohol and tobacco, and “netizens” with strong interest in free drinking at nightclub activities on various social media platforms. In addition, patients enrolling in drug rehabilitation programs were recruited as hospital seeds. Despite the variation in seed sources, subsequent respondents were recruited primarily on the basis of their substance-using network, without any confinement to the initial setting. From 2007 to 2010, a total of 47 seeds who resided in a variety of geographic regions in Taipei were recruited (35 seeds from the community sites and 12 seeds from hospital drug rehabilitation programs).

#### Recruitment rule

The criteria for respondent recruitment were (i) aged between 18 and 50 years, (ii) current user of both tobacco and alcohol, and (iii) current resident of metropolitan Taipei. Each recruited individual was asked to refer up to five of their recently contacted friends who regularly used both tobacco and alcohol, but most of them only referred one to three. A convenience store coupon with a face value of New Taiwan Dollars (NTD)$300 (approximately United States Dollars [USD]$10) was offered upon completion of the interview, and another coupon of NTD$100 was further offered for each successful referral. The recruitment phase was repeated until equilibrium was attained (about another eight waves).

#### Information on social network

A respondent who completed the interview (ie, became a recruiter) was asked to contact friends (ie, recruits) who fit the recruitment criteria to participate in the study. If recruits agreed to join our study, they could contact us directly. Interviewers then made an appointment with the referred recruits individually and set up the interview at locations of their convenience, including school campuses, companies, private homes, or nearby public places (eg, coffee shops). All recruits were asked about the nicknames of their recruiters and their relationships to verify and subsequently delineate their network structures. Social relational coding was used to mark their network relationships and the order in the referral chains, with the first four columns denoting the seed number and the subsequent columns denoting the wave. For example, a seeded individual was coded ‘F001-0000000000’. The first-wave and second-wave recruits were coded as ‘F001-1000000000’ and ‘F001-1100000000’, respectively. Before the interview, a safe place (eg, street corner) was chosen for respondents to sign an informed consent with a nickname. Participants were reassured of confidentiality before and during the interview.

The sample size needed was estimated to be about 500 or more according to the literature.^5^^,^^30^ However, due to budget constraints, the RDS was carried out on a yearly basis, rather than in a single implementation, using the same guidelines each year. The numbers of participants recruited in this fashion were 144 in 2007, 328 in 2008, 350 in 2009, and 293 in 2010. The aggregated sample size of the whole RDS implementation was 1115.

### Measurement

All information concerning social demographic characteristics, risk-taking behaviors (eg, substance use frequencies and patterns and sexual behaviors), family history of illegal drug use, and attitudes toward illegal drug use was collected via audio computer-assisted self-interview (ACASI). For those with any illegal drug experience, further inquiries were made regarding age at first use, the first use setting, and treatment-seeking behaviors.^17^^,^^31^ Using the ACASI, participants’ responses were saved immediately and invisibly on the computer screen in the interview, thereby ensuring confidentiality. In the present study, the assessment for illegal drugs included club drugs (ie, marijuana, ecstasy, and ketamine) and hard drugs (ie, methamphetamine and heroin).

### Statistical analysis

To assess the required number of recruitment waves and the prevalence of illegal drug use in this RDS study, equilibrium and the estimates were evaluated using the RDS Analysis Tool (RDSAT) software (Cornell University, Ithaca, NY, USA).^32^ We judged a recursive process to have reached equilibrium when the discrepancy in the sample proportion of a drug use between two adjacent waves was consistently less than 2%. This is in keeping with the default setting of RDSAT,^33^ in which a 5% deviation is thought to be indicative of equilibrium.

To derive estimates of prevalence, RDS assumes that the transition (or referral) probabilities within a population’s network depend only on the characteristics of the referring participant to form a Markov chain. When the transition probability reaches a steady state after several waves of referral, there is a probability that a participant with one characteristic (eg, ketamine use) will refer a participant with another characteristic (eg, without ketamine use). This transition probability is used as the individualized weight to adjust the crude sample proportions and produce the estimates of prevalence. Thus, in addition to raw sample-based proportion (ie, crude prevalence), lifetime and past-year prevalence of participants’ substance use was obtained using two different methods: equilibrium-based proportion (ie, an estimate derived from the transition probability at equilibrium) and RDS-adjusted population proportion (ie, an estimate using both transition probability and the network weighting system). Standard errors were estimated via the bootstrapping method. The Wald test was used to examine the differences in the prevalence between the two subgroups of early-onset and late-onset smokers.

## RESULTS

The distributions of sociodemographic characteristics of the total sample recruited via RDS are displayed in Table 1. Briefly, more than half of the participants were male (57.3%), two-thirds were less than 30 years old (66.2%) and had not attained a college degree (67.3%), over 60% were employed and had a monthly income less than NTD$30 000 (about USD$1000), and 1.1% of the participants reported being infected with HIV. When the sample was stratified by the initiation age of tobacco smoking (right panel of Table 1), individuals with onset age <18 years were more likely to be male, aged <30 years, less educated, and students, and they were less likely to be employed than those with onset age ≥18 years. However, the distributions of income and physical illness were similar between the two strata. One reason that individuals with onset age <18 years were more likely to be students and less likely to be employed but had no difference in monthly income than those with onset age ≥18 years was that some students who initiated tobacco use before age 18 were in vocational school and got paid through their internships or had part-time jobs.

**Table 1.** The distributions of socio-demographic characteristics and physical illness of the respondents recruited via RDS in Taipei, with stratification by the onset age of tobacco smoking

Variable	Total sample (*n* = 1115)			Initiation of tobacco smoking <18 years (*n* = 647)			Initiation of tobacco smoking ≥18 years (*n* = 468)			*P*-value^e^
		
	*n*^a^	%_wt_^b^	(95% CI)	*n*^a^	%_wt_^b^	(95% CI)	*n*^a^	%_wt_^b^	(95% CI)	
Male gender	689	57.3	(51.2–63.1)	450	66.7	(60.0–72.5)	239	44.9	(36.9–53.4)	<0.001
Age <30 years	737	66.2	(60.1–71.9)	455	70.4	(63.5–76.4)	282	61.0	(52.8–69.4)	0.001
Education <college	701	67.3	(62.7–72.8)	461	73.0	(67.7–79.5)	240	59.9	(52.6–67.9)	<0.001
Employment status										
Student^c^	345	29.0	(23.6–34.3)	214	33.4	(25.9–40.0)	131	24.3	(18.0–30.8)	<0.001
Employed^d^	691	62.3	(56.4–68.0)	381	56.5	(49.5–64.4)	310	68.7	(61.8–75.8)	<0.001
Unemployed	79	8.7	(5.9–12.1)	52	10.1	(6.3–15.1)	27	7.0	(3.5–10.8)	0.064
Monthly income (NTD$)										
≤10 000	182	19.9	(14.5–23.1)	100	19.6	(13.3–24.4)	82	20.0	(12.6–24.9)	0.869
10 001–30 000	348	40.4	(34.1–46.2)	213	40.4	(32.9–46.6)	135	40.0	(31.5–51.0)	0.893
≥30 001	357	39.7	(35.3–47.5)	193	40.1	(34.2–49.6)	164	40.0	(31.6–49.3)	0.973
Illness										
Ulcer	40	4.0	(2.5–6.4)	26	4.5	(2.4–7.4)	14	3.2	(1.2–6.2)	0.259
Hepatitis	48	4.8	(3.2–7.5)	40	6.6	(4.2–10.5)	8	1.8	(0.3–4.3)	<0.001
HIV/AIDS	17	1.1	(0.1–1.9)	12	1.2	(0.2–2.4)	5	0.9	(0.0–1.6)	0.624
Other STD	10	1.0	(0.2–2.0)	6	1.0	(0.1–2.9)	4	1.0	(0.0–1.3)	1.000

AIDS, acquired immunodeficiency syndrome; CI, confidence interval; HIV, human immunodeficiency virus; STD, sexually transmitted disease.

^a^Seeds are included in the numbers.

^b^Weighted percentages and their 95% confidence intervals are RDS-adjusted population proportions estimated using the RDSAT software.

^c^Either as a full-time student or in a work-study program.

^d^Full-time or part-time job.

^e^Wald test for the differences in prevalence between those who started using tobacco before age 18 and those who started at or after age 18.

The proportion of respondents with drug use in the accumulated RDS sample at each recruitment wave for the five most commonly used illegal drugs (ketamine, ecstasy, marijuana, methamphetamine, and heroin) as well as their combinational use (polydrug use not involving hard drugs and polydrug use involving hard drugs) are displayed in Figure. Through estimation by the RDSAT software, the greatest number of recruitment waves needed to reach equilibrium for estimating the use prevalence was five for heroin and three for other drugs (ie, ketamine, ecstasy, marijuana, and methamphetamine). Hence, the actual number of recruitment waves in the RDS sample was larger than the number needed to reach equilibrium.

**Figure.**
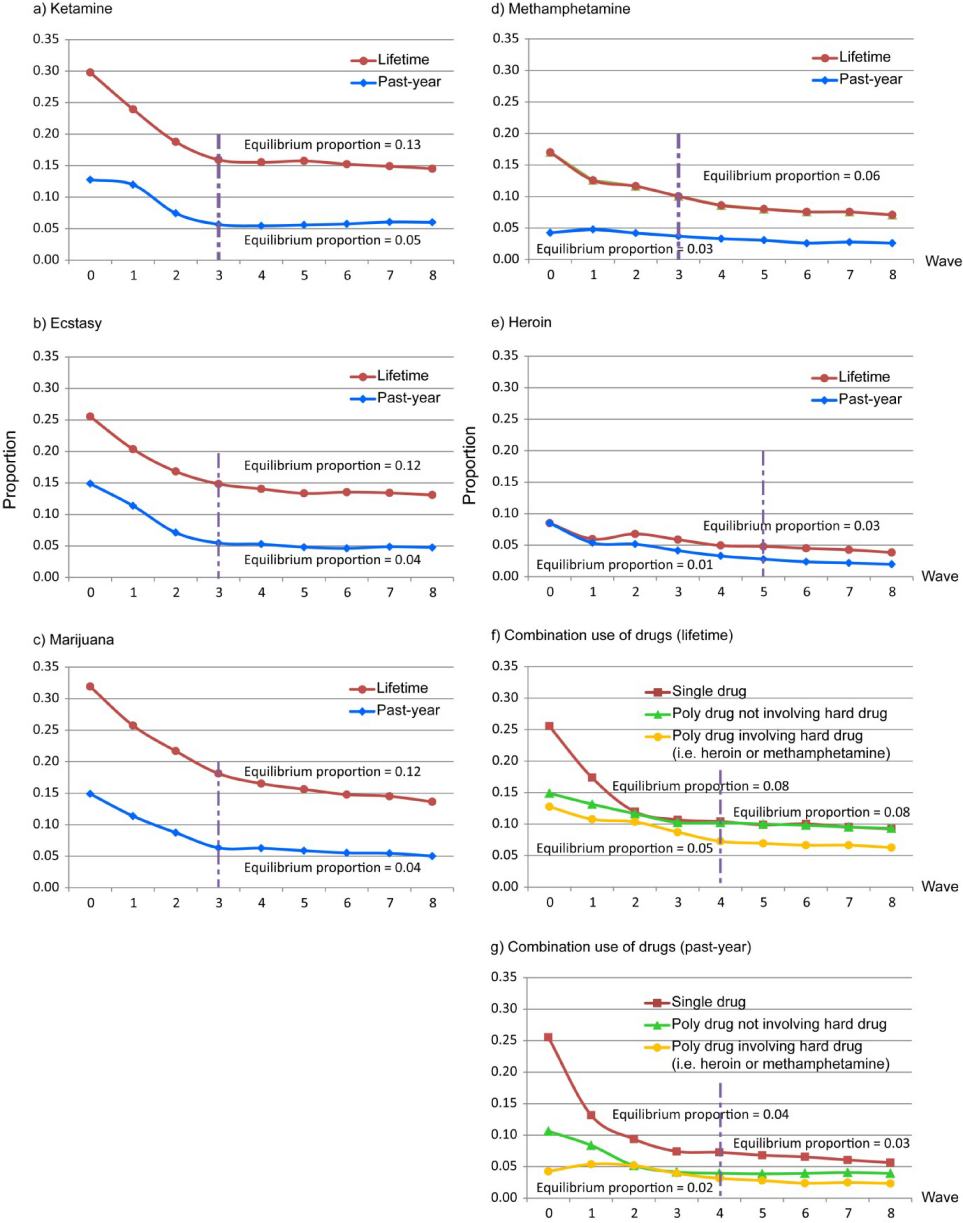
Proportions of lifetime and past-year use in the accumulated RDS sample at each recruitment wave for individual illegal drug use and combination use (single drug, poly drug not involving hard drugs [ie, heroin or methamphetamine], and poly drug involving hard drugs): a) ketamine; b) ecstasy; c) marijuana; d) methamphetamine; e) heroin; and f) poly-drug in the RDS sample (*n* = 1115). A vertical dashed line indicates the boundary beyond which the sample proportion started to converge to the equilibrium (ie, difference <0.02). The assessment of equilibrium was tested using software RDSAT.

In our RDS sample of 1115 regular tobacco and alcohol users, 277 had ever used illegal drugs in their lifetime. Table 2 displays the lifetime prevalence of the five most commonly used illegal drugs for the sample. The RDS-adjusted estimates for ever using ketamine, ecstasy, and marijuana were almost equally common (ranging from 11.7% to 10.5%), followed by methamphetamine (6.0%) and heroin (2.7%). For the whole sample, 21% of the RDS participants had ever used any illegal drugs, 18% had ever used any club drug (ie, ketamine, ecstasy, or marijuana), and 6.5% had ever used any hard drug (ie, methamphetamine or heroin). Regarding the drug use patterns, the lifetime prevalence of using a single drug (8.2%) was lower than that of polydrug use (13.8%), in which the figure of polydrug use not involving hard drugs (7.6%) was higher than that of polydrug use involving hard drugs (5.2%). When stratified by age at onset of tobacco smoking, the early-onset smokers were two to four times more likely to use illegal drugs than late-onset smokers, and the differences between these two strata were statistically significant in all drug categories, drug composites, and use patterns (all *P* < 0.01). For example, the ratio of the lifetime prevalence of ketamine use by early-onset smokers (16.3%) to late-onset smokers (5.7%) was 2.9 (*P* < 0.001).

**Table 2.** Lifetime prevalence of illegal drug use among the regular tobacco and alcohol users recruited via RDS in Taipei

Illegal drugs	Total sample (*n* = 1115)			Initiation of tobacco smoking <18 years (*n* = 647)			Initiation of tobacco smoking ≥18 years (*n* = 468)			Ratio	*P*-value^g^
		
	*n*^a^	%_wt_^b^	(95% CI)	*n*^a^	%_wt_^b^	(95% CI)	*n*^a^	%_wt_^b^	(95% CI)		
*Drug category*											
Ketamine	162	11.7	(9.3–14.8)	125	16.3	(13.0–21.5)	37	5.7	(3.3–8.6)	2.9	<0.001
Ecstasy	146	11.7	(8.8–14.9)	105	15.0	(11.2–19.6)	41	7.5	(3.8–11.1)	2.0	<0.001
Marijuana	152	10.5	(8.0–13.4)	104	13.6	(9.7–18.1)	48	6.4	(4.0–9.4)	2.1	<0.001
Methamphetamine	79	6.0	(3.6–8.5)	64	10.2	(6.6–14.8)	15	2.7	(0.9–4.9)	3.8	<0.001
Heroin	43	2.7	(1.0–4.6)	34	4.3	(1.7–7.9)	9	1.1	(0.2–2.8)	3.9	<0.001
*Drug composite*											
Any illegal drug	277	21.2	(17.3–25.0)	206	28.8	(23.7–34.1)	71	11.5	(7.3–15.8)	2.5	<0.001
Any club drug^c^	245	18.0	(14.6–21.3)	180	23.6	(19.1–28.4)	65	10.6	(6.6–14.5)	2.2	<0.001
Any hard drug^d^	85	6.5	(3.9–9.0)	70	11.1	(7.0–15.5)	15	2.7	(1.0–4.8)	4.1	<0.001
*Use pattern*											
Single drug	103	8.2	(5.9–10.3)	79	6.1	(4.1–7.6)	24	2.0	(0.8–3.5)	3.1	<0.001
Polydrug not involving hard drugs^e^	104	7.6	(5.7–10.3)	72	5.6	(4.0–8.0)	32	2.0	(1.1–3.1)	2.8	0.001
Polydrug involving hard drugs^f^	70	5.2	(2.9–7.4)	55	4.9	(2.9–7.4)	15	1.1	(0.3–1.9)	4.5	<0.001

CI, confidence interval.

^a^Seeds are included in the numbers.

^b^Weighted percentages and their 95% confidence intervals are RDS-adjusted population proportions estimated using the RDSAT software.

^c^Ketamine, ecstasy, or marijuana.

^d^Heroin or methamphetamine.

^e^Two or more illegal drugs but never used heroin or methamphetamine.

^f^Two or more illegal drugs and also used heroin or methamphetamine.

^g^Wald test for the differences in prevalence between those who started using tobacco before age 18 and those who started at or after age 18.

When prevalence was limited to past-year use (Table 3), the prevalence rankings for use of the five illegal drugs were similar to lifetime prevalence, with ketamine (4.3%), ecstasy (4.0%), and marijuana (3.8%) being the most commonly used drugs, followed by methamphetamine (2.9%) and heroin (0.9%). In terms of composite drug use in the past year, 8.9% of the RDS participants had used any illegal drugs, 7.0% had used any club drug, and 3.2% had used any hard drug. Regarding the drug use patterns in the past year, the prevalence of single-drug use was 4.5%, whereas that of polydrug use not involving hard drugs was 3.0% and that of polydrug use involving hard drugs was 1.8%. When stratified by age at onset of tobacco smoking, early-onset smokers also exhibited higher past-year use prevalence estimates of individual drug categories than late-onset smokers, though the difference only reached statistical significance for some categories (ketamine, methamphetamine, any illegal drug, any hard drug, and single drug use). In general, the ratios of early-onset smokers to late-onset smokers in the past-year use prevalence, regardless of individual drug categories, drug composite, or use pattern, were less than the corresponding ratios in the lifetime use prevalence.

**Table 3.** Past-year prevalence of illegal drug use among regular tobacco and alcohol users recruited via RDS in Taipei

Risk behaviors	Total sample (*n* = 1115)			Initiation of tobacco smoking <18 years (*n* = 647)			Initiation of tobacco smoking ≥18 years (*n* = 468)			Ratio	*P*-value^g^
		
	*n*^a^	%_wt_^b^	(95% CI)	*n*^a^	%_wt_^b^	(95% CI)	*n*^a^	%_wt_^b^	(95% CI)		
*Drug category*											
Ketamine	67	4.3	(2.8–6.2)	47	5.6	(3.5–8.6)	20	2.9	(0.9–5.0)	1.9	0.023
Ecstasy	53	4.0	(2.2–6.1)	32	3.8	(2.1–6.4)	21	4.1	(1.2–7.6)	0.9	0.800
Marijuana	56	3.8	(2.3–5.7)	33	4.7	(2.4–7.2)	23	2.7	(1.4–4.8)	1.7	0.074
Methamphetamine	29	2.9	(1.5–4.7)	22	4.4	(2.2–7.3)	7	1.9	(0.3–4.0)	2.3	0.015
Heroin	22	0.9	(0.2–2.3)	16	1.0	(0.1–3.1)	6	0.7	(0.0–2.0)	1.4	0.585
*Drug composite*											
Any illegal drug	130	8.9	(6.3–12.0)	90	11.1	(7.9–15.4)	40	6.4	(3.2–10.0)	1.7	0.005
Any club drug^c^	103	7.0	(4.8–9.5)	69	8.0	(5.3–11.2)	34	5.5	(2.5–9.1)	1.5	0.095
Any hard drug^d^	42	3.2	(1.7–5.4)	31	4.9	(2.6–8.4)	11	2.0	(0.5–4.0)	2.5	0.007
*Use pattern*											
Single drug	63	4.5	(2.7–6.6)	50	3.5	(2.2–5.3)	13	1.1	(0.2–2.4)	3.2	0.006
Polydrug not involving hard drugs^e^	44	3.0	(1.7–4.8)	26	2.0	(1.0–3.4)	18	1.0	(0.3–1.8)	2.0	0.163
Polydrug involving hard drugs^f^	26	1.8	(0.8–3.0)	17	1.2	(0.4–2.2)	9	0.7	(0.2–1.4)	1.7	0.385

CI, confidence interval.

^a^Seeds are included in the numbers.

^b^Weighted percentages and their 95% confidence intervals are RDS-adjusted population proportions estimated using the RDSAT software.

^c^Ketamine, ecstasy, or marijuana.

^d^Heroin or methamphetamine.

^e^Two or more illegal drugs but never used heroin or methamphetamine.

^f^Two or more illegal drugs and also used heroin or methamphetamine.

^g^Wald test for the differences in prevalence between those who started using tobacco before age 18 and those who started at or after age 18.

## DISCUSSION

This study demonstrates the practicality of RDS in estimating the prevalence of illegal drug use among individuals who were regular tobacco and alcohol users in Taiwan. Our results show important attributes of using RDS to recruit illegal drug users—a hidden population in social milieus. Asking each respondent to refer acquaintances using similar legal substances in his/her network might make potential recruits less wary of potential identification regarding their illegal drug use. These findings may support the application of RDS in epidemiological studies on illegal drug use.

This study provides us an opportunity to evaluate whether different illegal drugs need different numbers of waves in recruitment to reach a stable estimate. Most of the sample proportions of illegal drug use converged to equilibrium proportions around the third wave in our RDS sample, despite the seeds in our sample varying substantially in their socio-behavioral background and being recruited from different settings. Regardless of seed sources, the observation of equilibrium attainment may be partly a reflection of two important features of our RDS implementations. First, most of the respondents (95%) recruited only one to three of their friends or relatives. This may reflect that the effect of clustering among recruits was not as high as expected and the sampling process is instead a random walk on the network connecting the target population. Second, the recruiting chain lasted long enough to reach equilibrium (about eight waves), causing the prevalence estimate to converge to equilibrium. Both of these practices increase the chance of randomly recruiting peers with similar behaviors and thereby reduce potential selection bias.^4^^,^^30^

In the network of regular tobacco and alcohol users constructed via the RDS, 277 illegal drug users were recruited (about 21% of our study sample). For comparison, in 2 cross-sectional surveys in Taiwan, only 255 cases (1.7% of the sample of 13 168) in 2005 and 189 cases (1.4% of the sample of 14 192) in 2009 of the nationally representative samples aged 18 to 54 reported that they had ever used any illegal drugs.^28^^,^^29^ Having such a limited number of illegal drug users in the national surveys, it was difficult to reliably characterize epidemiological attributes of illegal drug users in the population. This indicates that RDS can be an efficient and effective method of reaching illegal drug users in Taiwan, where the prevalence of illegal drug use has been estimated to be low.

Among RDS respondents who had ever used any illegal drugs, most had ever used club drugs, and more than half of them had polydrug experience. These findings indicated that regular tobacco and alcohol use was highly comorbid with illegal drug-using behaviors, and a large body of alcohol and tobacco users had used more than one drug in their lifetime. These findings are consistent with previous studies showing tobacco and alcohol as gateways to more advanced drug use.^15^^,^^16^^,^^33^^–^^35^

Our results also highlight the differential drug-using behaviors between early-onset and late-onset tobacco smokers. Selling tobacco to young people aged less than 18 years is prohibited by law in Taiwan. Thus, smoking initiation before age 18 years is a deviant behavior among young people. This impression is matched with our findings that the RDS respondents who started smoking before age 18 years had greater risk of ever using illegal drugs (across the whole spectrum of illegal drugs) than those who started smoking from age 18 years. The finding implies that the earlier people start smoking, the more likely they are to use illegal drugs. This is consistent with the literature showing that people with early onset of tobacco smoking had greater risk of becoming tobacco dependent, more difficulty quitting smoking, and more frequent comorbid use of other psychoactive drugs subsequent to nicotine dependence.^18^^–^^22^

However, the prevalence estimates of past-year use of all five categories of illegal drugs were much closer between early-onset and late-onset tobacco smokers. For some drugs, such as ecstasy, the past-year prevalence rates were almost the same (about 4%) between the early-onset and late-onset smokers, while for other drugs, the differences of past-year prevalence between early-onset and late-onset smokers were relatively smaller than those of lifetime prevalence. RDS respondents’ current illegal drug-using behaviors might therefore be closely related to factors other than onset age of tobacco smoking, such as high-risk sexual behavior or having drug-using peers. A closer examination of the association between these factors and past-year illegal drug use is warranted.

This study has several limitations. First, aggregating data in different years for prevalence estimation may be questioned, since prevalence estimate might vary with time. However, due to limited funding and manpower, the sample size in each year was relatively small, and the prevalence of illegal drug use was stable in the repeated cross-sectional national surveys in 2005 and 2009. The data were aggregated for unbiased estimation, but the RDS-adjusted prevalence estimates based on our RDS samples pooled across years should be interpreted as the average prevalence estimates during the 4-year period. Second, whether or not the sampling from recruiters’ personal networks was random was difficult to evaluate in this study, because the characteristics of those who refused to be recruited were not collected. Therefore, we are unable to determine whether our results under- or over-estimated the true prevalences. Lastly, our RDS-adjusted estimates may be influenced by a masking effect. For example, some seeds with hard drug experience might recruit their legal substance-using colleagues rather than peers who also use hard drugs. Extension of a longer referral chain and use of more diverse seeds are needed to eliminate a potential masking effect. Given our insufficient funding and manpower, the recruitment was limited to eight waves; however, this was still three to five waves more than the number needed to attain equilibrium.

In conclusion, our results provided an empirical basis for the practical utility of RDS to obtain reliable epidemiologic estimates of illegal drug use in a population characterized by regular use of tobacco and alcohol. In this population, those who started smoking tobacco before age 18 were more likely to use illegal drugs and be polydrug users than those who started later in life. Given the recent rapid changes in technology and social behavior, network-based sampling (eg, RDS) and computer-assisted interviews might make it easier to approach participants from a wide range of backgrounds and recruit drug-using cohorts than with other modes of sampling methods (eg, random sampling).
